# Cardiotoxicity with vascular endothelial growth factor inhibitor therapy

**DOI:** 10.1038/s41698-018-0056-z

**Published:** 2018-05-08

**Authors:** Rhian M. Touyz, Joerg Herrmann

**Affiliations:** 1 Institute of Cardiovascular & Medical Sciences, BHF Glasgow Cardiovascular Research Centre, University of Glasgow, Glasgow, UK; 2 Department of Cardiovascular Diseases, Mayo Clinic, Rochester, MN USA

## Abstract

Angiogenesis inhibitors targeting the vascular endothelial growth factor (VEGF) signaling pathway (VSP) have been important additions in the therapy of various cancers, especially renal cell carcinoma and colorectal cancer. Bevazicumab, the first VSP to receive FDA approval in 2004 targeting all circulating isoforms of VEGF-A, has become one of the best-selling drugs of all times. The second wave of tyrosine kinase inhibitors (TKIs), which target the intracellular site of VEGF receptor kinases, began with the approval of sorafenib in 2005 and sunitinib in 2006. Heart failure was subsequently noted, in 2–4% of patients on bevacizumab and in 3–8% of patients on VSP-TKIs. The very fact that the single-targeted monoclonal antibody bevacizumab can induce cardiotoxicity supports a pathomechanistic role for the VSP and the postulate of the “vascular” nature of VSP inhibitor cardiotoxicity. In this review we will outline this scenario in greater detail, reflecting on hypertension and coronary artery disease as risk factors for VSP inhibitor cardiotoxicity, but also similarities with peripartum and diabetic cardiomyopathy. This leads to the concept that any preexisting or coexisting condition that reduces the vascular reserve or utilizes the vascular reserve for compensatory purposes may pose a risk factor for cardiotoxicity with VSP inhibitors. These conditions need to be carefully considered in cancer patients who are to undergo VSP inhibitor therapy. Such vigilance is not to exclude patients from such prognostically extremely important therapy but to understand the continuum and to recognize and react to any cardiotoxicity dynamics early on for superior overall outcomes.

## Introduction

Angiogenesis inhibitors have turned into clinical reality the pioneering vision of Dr. Judah Folkman’s that new blood vessel formation is critical for the growth of tumors and that anti-angiogenic therapy is key to tumor regression.^1^ Bevacizumab, a humanized monoclonal antibody directed against all isoforms of vascular endothelial growth factor (VEGF)-A, was the first targeted angiogenesis inhibitor to be developed. Since its approval in the US in 2004, it has emerged as one of the top ten best-selling drugs of all times, generating over US$60 billion in sales through 2016 (source: Forbes (1996 through 2012) and company-reported data from 2013–2016). World-wide, angiogenesis inhibitors approved for the treatment of malignancies have generated sales in excess of US$ 10 billion in 2014 alone (source: EvaluatePharma).

In patients with colorectal cancer and non-squamous cell lung cancer, the addition of the angiogenesis inhibitor bevacizumab doubled the progression-free survival. Similarly, in patients with metastatic renal cell carcinoma, sunitinib more than doubled overall survival over next line comparator therapy.^2^ The interested reader is referred to a recent review summarizing key Phase III clinical trial data for VEGF-inhibitors in advanced cancer.^3^ As testified, this class of drugs has emerged as a tremendous success story in health care.

On the other hand, adverse effects have been noted, including cardiovascular toxicities. These include both vascular, as well as cardiac side effects, which should not be a surprise based on the pivotal role of VEGF for the development and functional integrity of the vasculature and the importance of the vasculature for heart function. In this article we review the incidence, risk factors, and mechanisms of cardiac toxicity of angiogenesis inhibitors, namely those targeting the VEGF signaling pathway (VSP), and conclude with an outline of management options for clinical practice. The spectrum covered herein spans from hypertension to atherosclerosis, arterial thrombotic events, and heart failure. In particular, we aim to convey how the first three vascular toxicity profiles can ultimately culminate in cardiac disease. The content is based on a PubMed literature search covering the years 1960–2017 and using the search terms “angiogenesis inhibitor, arterial thrombotic events, atherosclerosis, cancer, cardiomyopathy, cardiotoxicity, chemotherapy, coronary artery disease (CAD), diabetes, heart failure, hypertension, hypothyroidism, obstructive sleep apnea (OSA), preeclampsia, vascular, VEGF, and VEGF inhibitor.”

## Cardiovascular events with VSP inhibitors

A number of cancer drugs, by virtue of their inhibitory effects on vascular growth signaling, can affect the survival and proliferation of endothelial and vascular smooth muscle cells and thus can exert an anti-angiogenic effect.^4^ However, no other growth factor signaling pathway has been as inherently entwined with angiogenesis as the VSP. Accordingly, VSP inhibitors are the epitome of this diverse class of drugs and will be the focus of this review (Table 1).

**Table 1:** FDA-approved vascular endothelial growth factor signaling pathway inhibitors

Drug (brand name)	Molecular targets	FDA approved for the treatment of
Aflibercept (Zaltrap)	Recombinant fusion protein of FLT-1 (VEGF receptor 1) and KDR (VEGF receptor 2) and immunoglobulin Fc component that captures (traps) VEGF-A, VEGF-B, and placental growth factor	Metastatic colorectal cancer
Axitinib (Inlyta)	c-KIT, PDGFR-A, PDGFR-B, FLT-1, KDR, FLT-4 (VEGF receptor 3)	Advanced renal cell carcinoma
Bevacizumab (Avastin)	Anti-VEGF-A antibody	Glioblastoma Persistent/recurrent/metastatic cervical cancer Metastatic colorectal cancer Non-small (nonsquamous) cell lung cancer Ovarian (epithelial), fallopian tube, or primary peritoneal cancer Metastatic renal cell cancer
Cabozantinib (Cabometyx Cometrig)	MET, KDR, FLT3, c-KIT, RET	Advanced renal cell carcinoma Medullary, locally advanced or metastatic thyroid cancer
Lenvatinib (Lenvima)	PDGFR-B, FLT-1, KDR, FLT-4, RET, c-KIT	Advanced renal cell carcinoma Advanced thyroid cancer
Pazopanib (Votrient)	ABL-1, c-KIT, PDGFR-A, PDGFR-B, FLT-1, KDR, FLT-4, FGFR, c-fms	Advanced renal cell cancer Advanced soft tissue sarcoma
Ramucirumab (Cyramza)	Anti-KDR antibody	Metastatic non-small cell lung Metastatic gastric Metastatic colorectal cancer
Regorafenib (Stivarga)	PDGFR-B, FLT-1, KDR, FLT-4, TIE2, RET, c-KIT, RAF	Metastatic colorectal cancer, locally-advanced, unresectable, or metastatic gastrointestinal stromal tumor, and hepatocellular carcinoma
Sorafenib (Nexavar)	B-Raf, FLT-1, FLT-3, FLT-4, KDR, KIT, PDGFR-A, PDGFR-B, FGFR, c-fms	Hepatocellular carcinoma Advanced renal cell cancer Differentiated thyroid cancer
Sunitinib (Sutent)	ABL-1, c-KIT, PDGFR-A, PDGFR-B, FLT-1, KDR, FLT-3, FLT-4, FGFR, SRC, c-smc	Gastrointestinal stromal tumor Pancreatic neuroendocrine tumors Renal cell cancer, adjuvant and advance
Vandetanib (Candetanib)	EGFR, KDR, FLT-4, RET	Medullary, locally advanced or metastatic thyroid cancer

Conceptually, the VSP can be inhibited on the level of the ligand or the extracellular or intracellular domain of the VEGF receptor.^5^ Pharmacologically two approaches are used to do so.^5^ The first entails monoclonal antibodies against the extracellular VSP components: VEGF isoforms (VEGF-A: bevacizumab and VEGF-A/VEGF-B/placental growth factor: aflibercept) and the VEGF receptor 2 (ramucirumab). The second approach comprises inhibitors of the tyrosine kinase activity of the intracellular domain of the VEGF receptor(s), which, however, is not as specific leading to diverse inhibitory profiles of (mainly receptor) tyrosine kinases.

Collectively, VSP inhibitors almost universally lead to an increase in blood pressure, the extent of which may vary, and depending on the blood pressure cut-offs used, various studies have reported varying levels of incidences. As outlined in a recent and most comprehensive meta-analysis of 77 VSP inhibitor studies, on average, severe hypertension is noted in 7.4% of patients, arterial thromboembolism in 1.8%, cardiac ischemic in 1.7%, and cardiac dysfunction in 2.3%.^6^ Overall, VSP inhibitors increase the odds of hypertension/severe hypertension, cardiac ischemia, arterial thromboembolism and cardiac dysfunction 5.3/5.6, 2.8, 1.5, and 1.4 times, respectively. A more tangible expression of risk, however, might be the number needed to harm, which is 6 and 17 for hypertension and severe hypertension, respectively. In comparison, 141 patients would need to be treated with VEGF inhibitor therapy for one arterial thromboembolic event to occur, 139 patients for one case of cardiac dysfunction to be noted, and 410 patients for one clinical heart failure (CHF) presentation to evolve. Cardiac ischemia may develop in 1 in 85 patients on VEGF inhibitor therapy, but the robustness of data is more limited as only eight studies were included for analysis of this endpoint (versus 71 studies for severe hypertension). Importantly, the risk of a fatal cardiovascular event with VEGF inhibitor therapy is very small, only 0.25%, or alternatively expressed, 1259 patients would need to be treated for one fatal event to occur. As such, it is important to emphasize that the risk-benefit balance of VSP inhibitors is heavily weighted toward using these agents rather than withholding them, especially as these agents are predominantly used in patients with metastatic malignancy, whose treatment options are limited.

## CAD as a risk factor for cardiotoxicity with VSP inhibitors

It stands to reason how much the cardiovascular events with VSP inhibitors are mechanistically related. Though only in a relatively small number of patients (*n* = 175), a very insightful study by Di Lorenzo et al.^7^ identified CAD and hypertension as the most important predictors for the development of heart failure with the VSP-TKI sunitinib. Importantly, all seven patients with a CAD history were among those 12 patients who developed CHF while on sunitinib. Accordingly, the positive predictive value of CAD for CHF with sunitinib was 100%, odds ratio 18.^7^ Even more, a study by Chu et al.^8^ identified CAD as the risk factor for CHF in patients treated with sunitinib with an odds ratio of 17 and for major adverse cardiac events (cardiovascular death, myocardial infarction, and CHF) with an odds ratio of 40 (three of the four patients with a CAD history developed CHD). These are strongly supportive data for CAD as a paramount risk factor for sunitinib cardiotoxicity but may apply to VSP inhibitors in general. The latter point may be supported by the higher relative risk for arterial thromboembolic events and cardiac ischemia in the aforementioned meta-analysis, which may translate into CHF, a point to be proven by correlative studies on patient level.^6^

The leading concept to explain the association of CAD with VSP cardiotoxicity relates to the concept of perfusion–contraction match, i.e., that myocardial contraction is coupled to and a reflection of myocardial blood flow (MBF) perfusion.^9,10^ This concept is utilized, for instance, in stress echocardiography testing.^11^ The reduction of myocardial perfusion though may not always be due to a focal coronary stenosis with a related abrupt drop in perfusion pressure.^12^ Rather, diffuse luminal diameter reductions over the epicardial course of the coronary arteries or several, by themselves not significant stenoses can generate additive effects sufficient enough to decrease perfusion pressure.^12^ Furthermore, abnormalities of the coronary microcirculation can increase the resistance to such a degree that impairment of perfusion of the myocardium evolves.^12^ VSP inhibitor therapy has the potential to contribute to all of these mechanisms, and patients with CAD have a conceptually lower margin of tolerability in this regard.

In a suitable rodent model marked acceleration of atherosclerosis was observed with a pan-VEGF receptor TKI.^13^ Increased plaque vulnerability, however, was not seen, which has been a theoretic concern for the use of VSP inhibitors.^14^ As such, this observation supports the theory that plaque neovascularization increases plaque vulnerability and anti-angiogenic therapy favors plaque stabilization.^14^ This being said, considering the paramount significance of VSP for vascular hemostasis, it is still not inconceivable that severe consequences can evolve from VSP inhibitor therapy, including endothelial cell apoptosis, plaque erosions, and acute arterial thrombotic events. In fact, very elegant basic science work confirmed the occurrence of all of these when autocrine VEGF signaling of endothelial cells was rendered insufficient.^15^

Before any of these structural phenomena evolve, VSP signaling inhibition can have profound functional implications. This relates to reduced nitric oxide production and the evolving endothelial dysfunction fosters arterial inflammation and atherosclerosis, platelet reactivity, and vasoconstriction.^16^ Superimposed on structural alterations, coronary vasoconstriction can lead to significant reductions in perfusion pressure and myocardial ischemia. The abnormal vasoreactivity of the coronary microvasculature may be even more profound in its effect.^17^ A major advance in this area was the discovery that sunitinib can significantly alter the integrity of the coronary microcirculation with evident reduction of the coronary flow reserve (CFR) and impairment of cardiac function.^18^ Intriguingly, inhibition of the platelet-derived growth factor (PDGF) signaling pathway seemed to be responsible for these phenomena, leading to depletion of the pericyte population, thereby destabilizing endothelial cells, the coronary microcirculation, and ultimately cardiac function. This has given recognition to the concept of pericyte–endothelial-myocardial coupling and the vascular nature of “cardiotoxicity” of VSP inhibitor therapy.^19^

At present it is unknown how these discoveries and concepts are best translated into clinical practice, if and how best to define any preexisting or evolving impairment of myocardial perfusion and the microcirculation that can then manifest in a decrease in cardiac function. Stress echocardiography could fulfill this role and was mentioned in the ASE consensus document as potentially being “helpful in the evaluation of patients with intermediate or high-pretest probability for CAD (echocardiogram uninterpretable or unable to exercise), who will receive regimens that may cause ischemia (fluorouracil, bevacizumab, sorafenib, and sunitinib).”^20^ Echocardiography can be expanded further to include myocardial perfusion assessment and even CFR.^17,21,22^ Otherwise, positron emission tomography (PET) and cardiac magnetic resonance imaging can be utilized for noninvasive quantification of regional MBF and MBF reserve.^17,23,24^ Cardiac catheterization would be the most direct approach, founded on coronary flow velocity (reserve) measurements, which along with coronary artery diameter measurements can be used to calculated coronary blood flow. Alternatively, the index of microvascular resistance can be calculated.^17^

Another aspect not clarified at this point is how soon VEGF inhibitor therapy can be resumed or started after an acute coronary syndrome/myocardial infarction. As a general timeline for the healing response, myocytolysis declines after 4 weeks and the risk of hemorrhage declines sharply after 2 weeks at a point in time when angiogenesis reaches a plateau.^25,26^ This corresponds to changes in the VEGF/VEGF-R expression profile. VEGF-A expression increases early after myocardial infarction, thereafter declining. VEGF-B expression, on the other hand, remains significantly suppressed and the expression of VEGF-C and VEGF-D is significantly increased, both early and late after myocardial infarction. VEGF receptor 3 expression is also increased, whereas the expression of VEGF receptors 1 and 2 is decreased. These findings suggest a functional role for VEGF-C and VEGF-D via VEGF receptor 3, which has traditionally been associated with the lymphatic system and may be involved in the repair process.^27^ There are no detailed studies looking specifically at this aspect of the VSP. One experimental study evaluating the effect of sorafenib in mice subjected to myocardial infarction did not find evidence of any alteration of post-myocardial infarction neovascularization or fibrosis.^28^ However, sorafenib led to apoptosis of cardiac- and bone-derived c-kit+ stem cells, thereby decreasing endogenous cardiac repair capacity. Intriguingly, beta-blocker therapy improved outcomes of sorafenib-treated mice; it ameliorated myocyte loss, improved cardiac function, and reduced mortality.^28^ It will be important to test whether these observations can be translated into the clinic and to define the field further. Beta-blocker therapy is standard of care for myocardial infarction patients already and timelines for starting or resuming VSP inhibitor after myocardial infarction may match the perioperative management guidelines, i.e., at least 60 days should pass from the acute myocardial infarction to subsequent stressors.^29,30^

## Hypertension as a risk factor for cardiotoxicity with VSP inhibitors

**Fig. 1:**
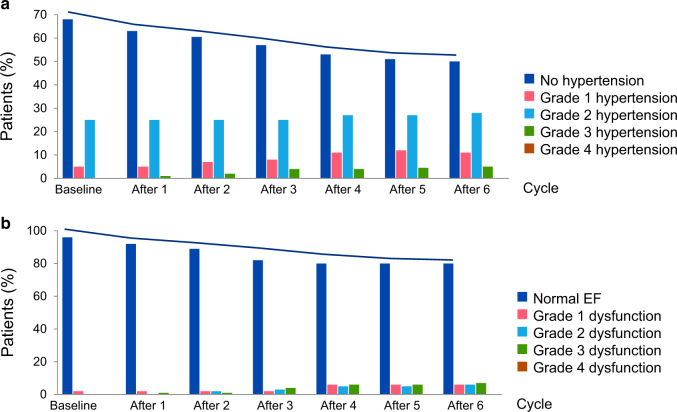
Hypertension and heart failure with sunitinib. Percentage of renal cell cancer patients free of hypertension and with various grades of hypertension (**a**) or heart failure (**b**) after initiation of sunitinib therapy. Modified from ref. ^7^

In addition to CAD, clinical studies on cardiotoxicity with sunitinib therapy outlined hypertension as a risk factor for heart failure.^7^ In fact, in the study by Di Lorenzo et al.^7^ all 12 patients who developed CHF or grade 3 cardiac dysfunction (left ventricular ejection fraction (LVEF) 20–39%) had systemic hypertension. In all but two cases the hypertensive response was of grade 3 degree (i.e., blood pressure >150 mmHg systolic or >100 mmHg diastolic with requirement of more than one antihypertensive or increase in the dosage of antihypertensive medication).^7^ Fourteen of the 17 patients developing grade 3 hypertension and nine of the 12 patients developing CHF/grade 3 cardiac dysfunction had systemic hypertension at baseline (all controlled, no patient had grade 3 hypertension before sunitinib). The development of grade 3 hypertension had a 70.6% positive predictive value and a 98.7% negative predictive value for the development of CHF. Of further interest, all patients had a normal LVEF at baseline, and all of them had a reduced LVEF at the time of presentation. As outlined in Fig. 1, the presentation of CHF cases evolved over the first three cycles, with one cycle typically being 4 weeks on and 2 weeks off, whereas the incidence of hypertension continued to rise. Blood pressure changes were most pronounced over the first three cycles, and the biggest increase in the incidence of grade 3 hypertension and grade 3 cardiac dysfunction was seen from cycles 2 to 3. This being said, the percentage of patients without hypertension continued to decline, whereas LVEF dynamics remained stagnant.^7^ On the contrary, the first comprehensive study on sunitinib cardiotoxicity in a subset of patients with the same treatment regimen noted a continuous, gradual decline of cardiac function despite stabilization of hypertension dynamics and initiation of beta-blocker and angiotensin converting enzyme inhibitor therapy in more than half of the cohort.^8^ Blood pressures were controlled to <140/90 mmHg, but compared with baseline values, they remained significantly elevated, raising the question whether control to baseline levels would be more beneficial. Taken together, while further studies are needed to define the relationship, hypertension may be considered as a significant risk factor for cardiotoxicity with VSP inhibitors and efforts to more aggressively control blood pressure may be rather beneficial. This is in keeping with the paradigm forwarded by the SPRINT trial and the most recent multi-societal guidelines on hypertension.^31,32^

**Fig. 2:**
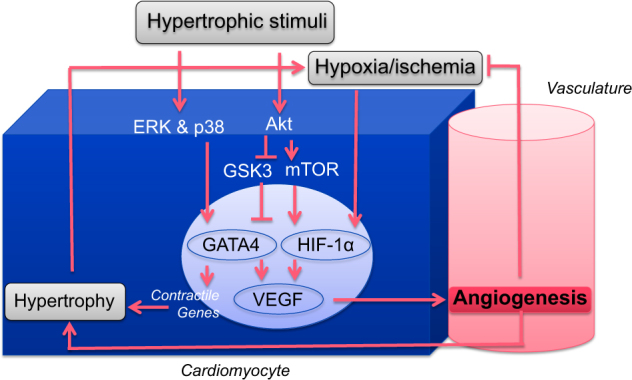
Myocardial hypertrophy and angiogenesis. Illustration of the VEGF-mediated, angiogenesis response to hypertrophic stimuli. Modified from ref. ^35^

In a large animal model, resting systemic blood pressure increases significantly within 1 week of starting sunitinib therapy in conjunction with an increase in systemic (and even coronary) vascular resistance.^33^ The potency of this change is underscored by a decrease in cardiac output occurring already at this stage in both large and small animal models without any evidence of abnormal myocardial vascularity and contractility.^33,34^ With chronic afterload increase, the heart aims to adapt by hypertrophic remodeling (Fig. 2). This hypertrophic response increases oxygen demand, stimulates the HIF-1a-VEGF axis and eventually leads to vascular neogenesis in the myocardium, aiming to meet the heightened metabolic demand.^35^ Izumiya et al.^36^ demonstrated not only cardiac hypertrophy on cardiomyocyte and organ level, but also an increase in cardiac expression of VEGF-A after thoracic aortic constriction (TAC) in mice, an established model of afterload increase. Interestingly, capillary density did not match the increase in myocardial area, leading to a relative deficit that was significantly increased with the expression of a VEGF receptor decoy protein. While fractional shortening was lower and left-ventricular end-diastolic pressure was higher with TAC alone, these reflections of cardiac dysfunction were much more pronounced in TAC animals expressing the VEGF receptor decoy protein. The noteworthy association of capillary density (anatomic microcirculatory reserve) with cardiac function is in keeping with the principle of perfusion–contraction match, which is not limited to the epicardial vasculature. Interestingly, these dynamics are recapitulated in mice treated with the VSP-TKI sunitinib and in patients treated with VSP inhibitors, who exhibit capillary rarefaction.^18,37^ The cardiomyocytes of these mice show mitochondrial swelling and degenerative changes, but apoptosis is not significantly increased unless sunitinib treatment is combined with an increase in blood pressure.^8^ A reduction in capillary density, of note, may not become apparent until combined with an increase in afterload, and afterload increase seems to be the common denominator for cardiac pathology to evolve.^18,38^ All in all these considerations are in support of optimal blood pressure control in the clinic.

Other prominent clinical examples where hypertension and cardiomyopathy are at play along with VEGF receptor decoy dynamics are preeclampsia and peripartum cardiomyopathy.^39^ In these women, soluble FLT-1 (sFLT-1), which is the non-membrane bound VEGF receptor 1 that can function as a decoy for VEGF, is secreted by the placenta in late gestation and significantly increased circulating levels are noted in women with peripartum cardiomyopathy, effects that are amplified when combined with preeclampsia. In a rodent model, exogenous sFLT-1 causes diastolic dysfunction and systolic dysfunction under circumstances of impairment of the metabolic and angiogenic reserve in the form of PPAR-gamma coactivator (PGC)-1α deficiency.^39^ Of note, PGC-1α is a transcriptional coactivator that is highly expressed in the heart and has been implicated in anthracycline cardiomyopathy as well.^40^ PGC-1α is important for antioxidant defense, mitochondrial homeostasis, and cell metabolism, as well as for the expression and secretion of pro-angiogenic factors, such as VEGF.^41^ PGC-1α deficient mice develop a dilated cardiomyopathy that is aggravated in the presence of sFLT-1.^39^ Not surprisingly, these mice respond poorly to stressful stimuli such as afterload increase by TAC and their survival is markedly reduced with increasing numbers of pregnancies, which also progressively decrease myocardial microvascular density.^39^ Importantly, providing VEGF rescues these mice from their detriment and confirms the vascular pathophysiology of peripartum cardiomyopathy. Very intriguingly, patients who recover fully from peripartum cardiomyopathy continue to have significantly higher circulating sFlt1 concentrations and significantly lower VEGF/sFlt1 ratios along with abnormal left ventricular (LV) global longitudinal strain values.^42^ Thus, the angiogenic imbalance persists in these patients along with subtle evidence of myocardial dysfunction, strengthening the view of their association.

## Conditions of increased afterload other than systemic hypertension as potential mediators/mechanisms of VSP-induced cardiotoxicity

The significance of the myocardial microvasculature and adaptive capillary growth for the structure and function of the myocardium is highlighted to another level in hypertrophic cardiomyopathy.^43^ Patients affected by this condition have reduced vascular capacity and CFR.^17^ This relates to the reduction of capillary density in correlation with the resting LV outflow tract gradient, on average by one third.^44,45^ In an experimental hypertrophy model, administration of recombinant VEGF promoted capillary growth and reduced cardiomyocyte apoptosis. It furthermore prevented cardiac dilation and contractility decline, delayed onset of heart failure, and prolonged survival.^46^

The outlined dynamics are also applicable to aortic stenosis, which potently induces cardiac hypertrophy over time, especially with a progressive increase in the outflow tract gradient. These patients likewise have a reduced CFR related to vascular rarefication.^17^ Very evident in adult male mice subjected to aortic stenosis, the increase in myocardial VEGF expression and capillary density lags behind the increase in cardiomyocyte area (hypertrophy) resulting in perturbed mitochondrial energetics, cardiomyocyte apoptosis, and a decline in cardiac contractility.^47^ Overexpression of VEGF in this setting boosts capillary density and mitochondrial energetics, reduces cardiomyocyte apoptosis, and preserves cardiac function without a change in cardiac mass or hypertrophy.^47^

Another condition associated with increased afterload is OSA.^48–53^ The apneic episodes in these patients cause hypoxia and carbon-dioxide retention sufficient enough to trigger ineffectual inspiratory efforts and the generation of negative intrathoracic pressure against the occluded pharynx. By increasing the difference between intracardiac and extracardiac pressure, LV transmural pressure increases, i.e., LV afterload.^54^ The negative intrathoracic pressure augments venous return and right ventricular (RV) preload, while hypoxia causes pulmonary vasoconstriction, increasing RV afterload.^54^ As a consequence, the pressure and the size of the RV increase with exaggerated interventricular dependence that reduces LV filling during diastole and decreases stroke volume during systole.^54^ These acute effects were confirmed in a canine model and animals chronically exposed developed cardiac hypertrophy and dysfunction.^55^ The combination of increasing afterload and hypertrophic stimulus on the one hand and hypoxemic episodes on the other hand is unique and may point to greater stimulation of compensatory angiogenesis via the HIF-1a-VEGF axis.^56,57^ Of interest, VEGF generation capacity in response to hypoxia correlates with collateral vessel formation capacity in CAD patients,^58^ and an impairment in this response translates into poorer clinical outcomes.^59–61^ Endothelial dysfunction and impaired myocardial perfusion is noted in otherwise normal subjects with moderate-to-severe OSA.^62^ Increases in sympathetic tone can further peripheral vasoconstriction and increase afterload. These dynamic effects of OSA may persist way into the awake period and can lead to sustained blood pressure elevations over time.^54^ As such OSA is not only strongly and independently associated with blood pressure elevation, but even resistant systemic hypertension and reinforces all of its consequences.^54,63^ Accordingly, when seeing patients for VSP inhibitor therapy it may be important to review not only whether systemic hypertension is present, but also which conditions could play a contributing role to and/or increase afterload beyond overt systemic hypertension.

## Hypothyroidism as a potential mediators/mechanisms of VSP-induced cardiotoxicity

In a review on “understanding and managing toxicities of VEGF inhibitors” Schmidinger forwarded the view that hypothyroidism induced by VSP-TKIs can contribute to their cardiotoxicity potential.^64^ Up to 85 and 21% of patients on sunitinib and sorafenib, respectively, develop subclinical or clinically overt hypothyroidism.^65^ Of note, both of these drugs have been used in the treatment of thyroid carcinoma owing to their potency to inhibit kinases involved in the growth and function of thyroid cells. VSP inhibitor-induced hypothyroidism is seen as a consequence of inhibition of the ret proto-oncogene product, suppression of iodine uptake and peroxidase activity, and induction of capillary regression.^64^ Regression of capillaries has been observed in a number of organs (especially endocrine organs) in adult rodents treated with VSP inhibitors.^66^ In the study by Chu et al.,^8^ five out of 36 patients (14%) on sunitinib developed hypothyroidism at a mean time of 54 weeks. While the incidence of heart failure in these patients was not reported, it was stated that the mean time to onset of heart failure was only half of this time interval and that CHF preceded hypothyroidism in all patients.^8^ On the other hand, one of the highest incidences of cardiotoxicity with VSP inhibitor is observed among patients with thyroid cancer.^67^ These patients are confronted with hypothyroidism as consequence of their therapy. Importantly, even subclinical hypothyroidism, low T3 levels, and borderline normal thyroid function can translate into impaired endothelial function (vasorelaxation), increased SVR, reduced cardiac output, and worse cardiovascular outcomes.^68–70^ A reduction in cardiac output is felt to be the consequence of a decrease in heart rate, cardiac filling (i.e., preload in addition to increased afterload), and cardiac contractility.^71,72^ Thus, while there is no sequential or otherwise direct evidence that hypothyroidism induced by VSP-TKIs is causal to the cardiotoxicity risk of these drugs, it remains important to diagnose and treat hypothyroidism in any cancer patient throughout the continuum of their care. The vascular and hemodynamic changes induced by clinical and even subclinical hypothyroidism are similar and presumably additive to those induced by VSP inhibitor therapy.^8,67,73–79^

## Diabetes mellitus as a potential mediator/mechanism of VSP-induced cardiotoxicity

Another condition associated with dysfunction of the coronary microvasculature and the development of cardiomyopathy is diabetes. A decline in cardiac function and progressive myocardial fibrosis over time has been demonstrated in experimental models of diabetes. This is associated with a concomitant decrease in myocardial capillary density and MBF.^80^ VEGF165 gene therapy by plasmid vector delivery counteracts these developments and provides a “cure.” As important as these observations are for the specific disease process, they also raise awareness that any additional interference with angiogenesis could be potentially more harmful for patients with diabetes. A progressive decline in CFR has, indeed, been observed in patients across the spectrum of diabetic disease severity that makes these patients more vulnerable.

In metastatic renal cell cancer patients VSP-TKIs were found to decrease blood glucose levels in both nondiabetics and diabetics.^81^ The effects can be profound, and diabetics may stop their medications. Inhibition of insulin clearance was found to be one mechanism, and hypoglycemic episodes can develop in patients using insulin or insulin-secretion stimulating agents. Other mechanisms discussed include capillary regression of pancreatic islets. Indeed, this has been observed in a mice, and sunitinib increased insulin sensitivity and peripheral glucose uptake.^82^ Sunitinib also increased glucose uptake in the heart 5-fold in conjunction with increased glucose metabolism and caused upregulation of enzymes of the glycolytic pathway, particularly the M2 isoform of pyruvate kinase, reflecting activation of the fetal gene program.^82^ These dynamics are enhanced by HIF-1 alpha activation, and thus aggravated by ischemia/hypoxia, either due to reduced blood supply or increased demand in the setting of hypertrophic disease processes. Of note, diabetic hearts are characterized by reduced glucose and glycolysis and enhanced fatty acid metabolism and it has been discussed that altered myocardial substrate and energy metabolism may contribute to the development of diabetic cardiomyopathy.^83–89^ One may thus argue that the pro-glycolytic action of sunitinib may be beneficial. However, two recent reports confirm that sunitinib increases cardiac glucose uptake and reliance on glycolysis, but there is failure to use glucose as an energy substrate (similar to insulin resistance and diabetic states). Even more, there is evidence of reduced oxidative phosphorylation, increased myocardial lipid deposition, and perturbed mitochondrial function, in keeping with the view of that sunitinib induces a fundamental energy crisis that results in compromised myocardial energy metabolism and function.^90,91^

In this context, the inhibitory effects of sunitinib on AMP kinase described previously gain further significance.^92–94^ This enzyme senses the energy state of cells and responds to increases in AMP:ATP and ADP:ATP ratios, reflecting energy rundown or energy stress, with activation of catabolic pathways that generate ATP.^95^ This includes the stimulation of glucose uptake and glycolysis, as well as oxidative phosphorylation and PGC-1 alpha, the significance of which was mentioned in the context of peripartum cardiomyopathy before. AMP kinase also has an important role for the stimulation of autophagy as an important process for organ viability. Under normal conditions, the significance of AMP kinase in controlling these processes is not as evident, but under ischemic stress conditions, deficiencies in these domains translate into a larger extent of myocardial injury/infarction and poorer recovery of cardiac function.^96^ These observations also provide an illustration of the potential impact of impairment of AMP kinase activity by sunitinib.^92–94^ Interestingly, metformin has been shown to enhance AMP kinase activity with cardioprotective effects in experimental models of ischemia/reperfusion injury, myocardial infarction, and anthracycline cardiotoxicity.^97–103^ Metformin has also been noted to be of benefit in dilated cardiomyopathy and prevents hypertrophy, which is of particular interest in the context of VSP inhibitors.^104–112^ If and how metformin could be utilized to counteract sunitinib cardiotoxicity or cardiomyopathy related to VSP inhibitor use in general and in patients with diabetes in particular is unknown but the hypothesis-generating data presented here would support such studies.^8,64,65,67,73–79^ Additional studies are also needed to define the risk equation for diabetic patients undergoing VSP inhibitor more accurately. Yet for now, conceptually it does seem prudent to error on the side of caution and to be considerate of their cardiovascular adverse event potential.

## Chemotherapy as a potential mediator/mechanism of VSP-induced cardiotoxicity

Another aspect to be considered is preceding or concomitant cancer therapy, potent enough to exert injury to the heart and/or its vasculature. Indeed, this occurs with various chemotherapeutics and radiation therapy to the chest. Again, inhibition of the VSP at a time when its activation is needed for recovery from injury can be detrimental. An example is provided in the MAIN trial, which randomized patients with diffuse large B-cell lymphoma to R-CHOP therapy with placebo or bevacizumab (10 mg/kg q 2 weeks or 15 mg/kg q 3 weeks). The trial was stopped after enrollment of 787 patients by the Data and Safety Monitoring Board because of a nearly 3-fold higher risk of drop in LVEF and heart failure in the absence of any therapeutic benefit.^113^ Given the multidrug nature of the R-CHOP regimen (Rituximab, Cyclophosphamide, doxorubicin hydrochloride (Hydroxydaunorubicin), vincristine sulfate (Oncovin), and Prednisone), the nature of the most significant interaction remains elusive.

Cyclophosphamide, for instance, can induce endothelial injury and apoptosis and thus has clear interaction potential with VSP inhibitors as does vincristine. Doxorubicin alters paracrine VEGF signaling in the myocardium, upregulating VEGF-A release from cardiac microvascular endothelial cells (CMECs) but decreasing VEGF receptor 2 expression in both CMECs and adult ventricular myocytes.^114^ In a cell culture model, (over-) expression of VEGF-A_165_ was found to protect cardiomyocytes from doxorubicin-induced apoptosis, related to upregulation of the Akt pro-survival signaling pathway.^115^

Another in vivo study showed that VEGF-B_186_ also exerts cardioprotective effects in mice subjected to doxorubicin.^116^ VEGF-B_186_ protected not only cardiomyocytes from injury, but also endothelial cells and the myocardial microvascular network. The potency of the VEGF-B_186_ effect on endothelial cells is underscored by its capacity to preserve endothelial function, a very sensitive marker of endothelial health. This beneficial effect was related to the robust expression of VEGF receptor 1 on endothelial cells in the myocardium.^117^ Of note, following myocardial infarction, prolonged intramyocardial expression of VEGF-A_165_ and VEGF-B_167_ preserves viable cardiac tissue, prevents ventricular remodeling, and results in improved cardiac function over time. The increase in contractile myocardium was more pronounced after expression of VEGF-B, even in the absence of significant induction of angiogenesis. VEGF-B signals through the VEGF-R1 receptor, which was upregulated under conditions of hypoxia and oxidative stress and elicited a particular gene expression profile of the compensatory, hypertrophic response, both in cultured cardiomyocytes and in infarcted hearts. This translated into powerful anti-apoptotic effect of VEGF-B in cardiomyocytes and mouse hearts in vivo.^118^ Cardioprotective effects of VEGF-B_167_ unrelated to angiogenesis were also confirmed in an experimental (pacing-induced) non-ischemic dilated cardiomyopathy model.^119^ VEGF-B did not change microvascular density but reduced the number of apoptotic cardiomyocytes in the heart, preserved systolic and diastolic cardiac function, and prevented LV wall thinning.

In cultured rat neonatal cardiomyocytes exposed to angiotensin II, VEGF-B_167_ prevented oxidative stress, loss of mitochondrial membrane potential, and, consequently, apoptosis.^119^ Thus, in addition to VEGF-A and the VEGF recptor-2, which has been the focus of the beneficial effects and side effects of VSP inhibitors, the above findings provide a different perspective. VSP inhibitors with an additional inhibitory effect on the activity of VEGF receptor 1 may therefore bear a higher risk of cardiotoxicity. While this may hold true for sunitinib in some studies, this has not been confirmed on a broader scale. Neither has pazopanib, which has additional anti-VEGF receptor 1 inhibitory properties, nor the pan-VEGF receptor TKI axitinib, been associated with a higher incidence of cardiotoxicity. Moreover, in preclinical screening studies, the therapeutic window of VSP antagonists was fairly comparable and not influenced by VEGF receptor 1 inhibiting properties.^120^ One may therefore conclude that the VEGF-B-VEGF receptor 1 axis is of benefit if stimulated, but its inhibition may not be detrimental per se. For the interaction with other chemotherapeutics, the most important consideration is the very likely additive adverse action on endothelial cells. While VEGF is expressed even in the normal myocardium, the consequences are most likely revealed when its expression is upregulated as part of a healing or compensation response, and it is under such circumstances that most cases of cardiotoxicity occur. The optimal time interval (if any) between the administration of VSP inhibitors and other vaso- and/or cardiotoxic agents remains to be defined. Overall, it is advisable to consider all possible multiple hits in any given cancer patients, which collectively may reduce the cardiovascular reserve (over time).^121^

## Unique aspects of VSP inhibitor cardiotoxicity

The healthy myocardium expresses all isoforms of VEGF and all three VEGF receptor subtypes. While VEGF-B, -C, and –D have generated some interest, the focus of VSP inhibitor therapy is still on VEGF-A and its main angiogenic receptor VEGF receptor 2. As outlined, this axis is stimulated by hypoxia and ischemia, as well as stretch in the heart, i.e., afterload increases, such as hypertension, both of which are risk factors for cardiotoxicity with VSP inhibitor therapy.^122^ The significance of this pathway has been delineated in conditional knockout mice in which VEGF expression is reduced in a cardiac-specific manner. Such intervention translated into a deceased microvascular density, as well as a suppressed cardiac function and reserve in keeping with the perfusion–contraction paradigm.^123^ Cardiomyocytes release VEGF upon exposure to various stressors, which then acts on microvascular endothelial cells in a paracrine fashion.^15^

Importantly, however, there is also auto-/intracrine signaling, i.e., the microvascular endothelial cells themselves express VEGF, which is of utmost significance for their survival. In fact, in the absence of auto-/intracrine VEGF, paracrine sources are not sufficient to sustain endothelial cell survival.^15^ Of further significance then, the action of VSP inhibitors acting on the extracellular domain of the VEGF receptor, i.e., the monoclonal antibodies can be overcome, whereas this is not the case for those acting at the intracellular sites of the receptor, such as VSP-TKIs. The actions of the latter drugs can thus be more profound. The potential for harm may be even greater the lower their target specificity. This has been suggested by the results of myocyte cell culture studies.^124^ Such promiscuity may extend to other receptor tyrosine kinases.

**Fig. 3:**
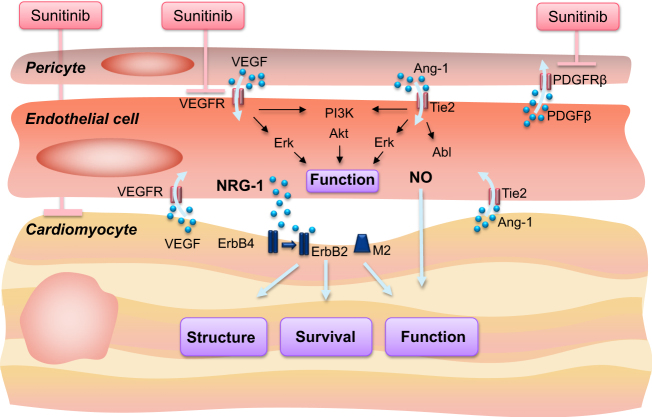
Pericyte–endothelial–myocardial coupling. Illustration of pericyte–endothelial–myocardial coupling. None of these cells exists in isolation in the myocardium, but there is interaction on multiple levels. Accordingly, cardiomyocytes can be affected by an initial action on endothelial/pericyte level. This has been shown for sunitnib, although it has been reported to affect all three outlined cell types

For instance, as mentioned above, sunitinib inhibits PDGFR receptor-ß tyrosine kinase as well. This translates into reduced myocardial pericytes, decreased myocardial microvascular density, and a further reduction in cardiac function when mice were subjected to sunitinib in addition to TAC.^18^ These findings highlight the importance of pericyte-endothelial-myocardial coupling (Fig. 3) and the significance of the integrity of the vasculature for cardiac function and reserve and help explain some reports on the association of cardiac troponin (cTn) elevation with the use of VSP-TKIs but not bevacizumab or mTOR inhibitors, even though all induce a comparable degree of hypertension and cardiac dysfunction.^125^ However, even though incidences may vary between the different agents and across different populations, comprehensive studies on this subject were unable to conclude that the (relative) risk of cardiotoxicity is higher with one type of VSP inhibitor than another and neither do meta-analyses describe notable differences in cardiovascular risk between direct VEGF inhibitors and small molecule agents.^6,126–130^ Thus, regardless of the extracellular mode of action on VEGF-A (bevacizumab) or intracellular mode of action on various VEGF subtypes, either selective to all VEGF receptor TKIs (axitinib) or non-selective to VEGF receptor TKIs and other TKI targets (e.g., sorafenib, sunitinib, and pazopanib), there are specific characteristics inherent/generic to VSP inhibition.

In a mouse model, treatment with bevacizumab led to a similar increase in blood pressure, drop in LVEF, extent of LV dilation, circulating cTn levels, and ultrastructural changes of the myocardium than treatment with sunitnib.^131^ Of note, the increase in blood pressure preceded the development of cardiotoxicity by 1 week. No interventions were performed to evaluate if any antihypertensive action would have changed the cardiac outcomes. The study, however, did make the point that any type of VSP inhibition, including VEGF-A directed therapy, has cardiotoxic potential.

**Fig. 4:**
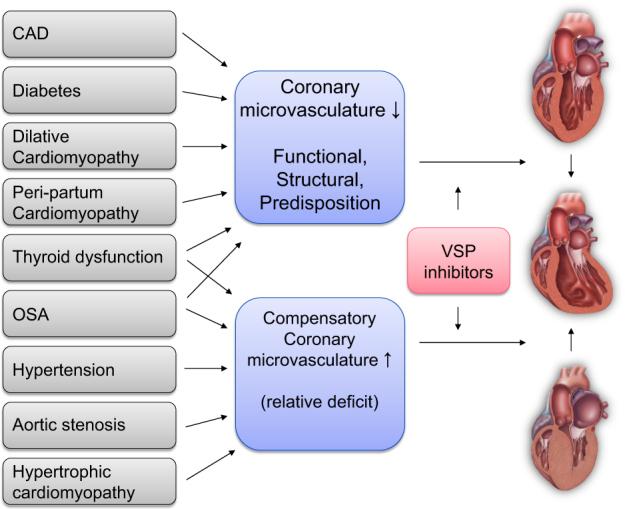
Conceptual outline of the vascular nature of VSP inhibitor cardiotoxicity. Outline of the concept of absolute or relative and structural or functional coronary microvascular deficit and cardiomyopathy with VSP inhibitor therapy

**Fig. 5:**
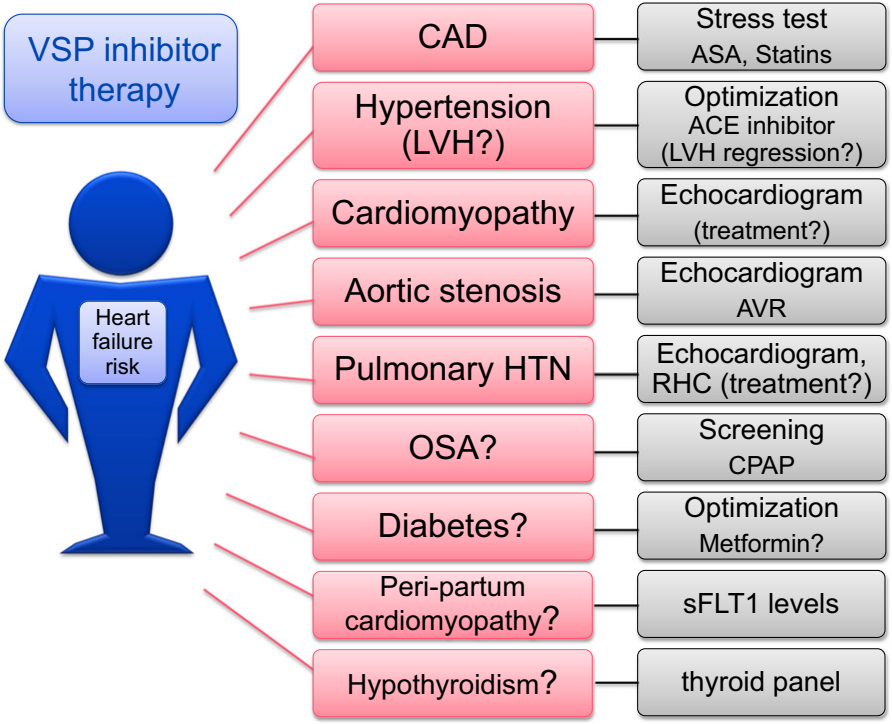
Comorbidities contributing to the vascular nature of VSP inhibitor cardiotoxicity. Illustration of the comorbidities to consider and to screen for in patients who are considered for VSP therapy matching the pathophysiological concept introduced in Fig. 4

## Monitoring and treatment of cardiotoxicity with VSP inhibitors

An important feature of VSP inhibitor-induced cardiotoxicity is its reversibility in most but not all (60–80%) of patients upon withdrawal.^7,132,133^ Indeed, prompt recognition and cessation of VSP inhibitor therapy is the single most important step to recovery, and the institution of classic heart failure therapy has been viewed to be more of an ancillary than crucial element.^7,133^ Cessation of VSP inhibitor treatment, however, is suboptimal from a cancer therapy perspective, and balancing the benefits and risks of VSP inhibitor cancer therapy is important yet challenging.

VSP inhibitor-induced cardiotoxicity is considered to be reminiscent of that observed with the HER-2 inhibitor trastuzumab. It differs from classical anthracycline cardiotoxicity not only by its reversible nature, but also by its lack of dose response and lack of influence of subsequent stressors and tolerance of reexposure. As outlined in the 2014 consensus documents of the American Society of Echocardiography and the European Association of Cardiovascular Imaging on multimodality imaging of adult patients during and after cancer therapy,^20^ patients undergoing VEGF inhibitor therapy are to be monitored every 3 months for the time of treatment with 2D (with contrast) or 3D echocardiograms. In distinction from other cancer therapeutics for which a similar type of cardiotoxicity has been described, the first follow-up evaluation of cardiac function should be one month after therapy with VSP inhibitors as cardiotoxicity can emerge sooner and sometimes very early on. Such dynamics are likely the reflection of the state of “angiogenic dependence” of the myocardium.

The 2014 ASE/EACI consensus document recommendations include strain imaging and cTn assessment in the algorithm of assessment 1 month and then every 3 months after start of therapy. One of the most comprehensive initial evaluations of sunitinib cardiotoxicity found that while 8% of the 75 patients enrolled developed heart failure, 80% of patients experienced some degree of LVEF decline (≥10 and ≥15% in nearly 30 and 20% of patients, respectively).^8^ The LVEF dynamics, however, were assessed only for 36 of these patients who had been on 50 mg of sunitinib per day, 4 weeks on and 2 weeks off. Based on repeated-measures, mixed-model regression analysis, it was predicted that LVEF would drop by 2% with the first and 1.5% with subsequent cycles. However, marked interindividual differences exist supporting close follow-up recommendations, and as the median time for heart failure was 31 weeks (range 11–85 weeks), over a prolonged period of time. Only one study has been published so far reporting on strain imaging in these patients. Global longitudinal strain decreased within 2 weeks of initiation of treatment and continued to decline further in those maintained on therapy.^134^ This was in the absence of any early change in LVEF, but how predictive it is for future LVEF drops is not known.

With regards to cTn, a similar-sized study of 74 consecutive patients treated with sunitinib or sorafenib for mRCC noted cTnT elevation in nine patients.^135^ In all but two of these patients the cTnT values were <0.1 ng/mL. Two thirds of the elevations were associated with ECG changes, slightly less than half had a reduced LVEF. Vice versa, four of the seven patients with a reduce LVEF and six of the 12 patients with ECG changes had cTnT elevation. Data on reversibility of cardiac dysfunction were not provided, but in all but one case VSP inhibitor therapy could be resumed, suggesting that cTnT elevation may not be a marker of irreversible cardiotoxicity in these patients. In agreement, another prospective study on 90 patients undergoing therapy predominantly with a VSP inhibitor found that ten patients developed chest pain, two of which had cTnI elevation, and eight had asymptomatic cTnI elevation. An abnormal LVEF and T-wave inversion were seen only in one patient each. No patient showed late gadolinium myocardial enhancement on cardiac magnetic resonance or had stenoses >50% on coronary angiography. All patients with either chest pain or cTnI elevation received beta-blocker and aspirin and were then re-challenged with the study drug without recurrent cardiovascular events.^135^ Thus, in patients on VSP inhibitor therapy cTn is not a sensitive marker for either the development or the irreversibility of cardiac dysfunction (“cardiotoxicity”).

One aspect not taken into consideration in any recommendation on monitoring and managing of cardiovascular toxicities with VSP inhibitor therapies is the evaluation and treatment of any potentially underlying and contributing comorbidity (Figs. 4 and 5). It is important that VSP inhibitor is not considered the culprit by default, even though it likely has a contributing role in the majority of cases. The real question in clinical practice often is whether it is the only factor or whether it unmasks an underlying disease state and the “angiogenic demand” of the patient. In the latter scenario, rechallenge may not be well tolerated until the underlying disease process is treated. Another important aspect is the quality of blood pressure management. Tighter blood pressure control, possibly with ambulatory and home blood pressure monitoring, may be helpful in this regard. Moreover, in patients with OSA, proper CPAP treatment may reduce the risk of VSP inhibitor-induced cardiac disease.

## Conclusions

A decline of cardiac function can occur in a subset of patients on VSP inhibitor therapy. To date, clear guidelines are lacking on how to risk-stratify and manage these patients. With increased evidence-based studies, however, comprehensive guidelines should become available to help diagnose and better manage VSP inhibitor-induced cardiovascular disease. In this review, we aimed to provide a conceptual framework that will help to understand and stimulate more studies on the pathophysiology of VSP inhibitor cardiotoxicity. Knowing the pathomechanisms and conditions will allow treating patients with VSP inhibitor therapy with a more precise understanding of the risk and mitigation strategies for cardiotoxicity. While acute fatal outcomes are rare, cardiotoxicity can carry a high burden of morbidity and can impair long-term outcome. Pro-actively recognizing and managing vascular and overall health issues of patients undergoing cancer therapy with VSP inhibitors is precisely for this: to enable patients to undergo such prognostically extremely important therapy uncoupled from the risk of cardiovascular diseases.
